# Exploring the Bioactive Compounds of *Haematococcus pluvialis* for Resistant-Antimicrobial Applications in Diabetic Foot Ulcers Control

**DOI:** 10.5812/ijpr-161297

**Published:** 2025-09-24

**Authors:** Fateme Mirzajani, Narges Parniaei, Faeze Mirzajani, Arefe Ghaderi

**Affiliations:** 1 Faculty of Life Sciences and Biotechnology, Shahid Beheshti University, Tehran, Iran; 2 Protein Research Center, Shahid Beheshti University, Tehran, Iran; 3 Department of Medical Laboratory Science, College of Science, Knowledge University, Erbil, Iraq; 4 Department of Agriculture and Food Science, Science and Research Branch, Islamic Azad University, Tehran, Iran

**Keywords:** Bacterial Inhibition, Diabetic Foot Ulcer, *Haematococcus pluvialis*, *Staphylococcus aureus*, Wound Healing

## Abstract

**Background:**

Diabetes is a contemporary ailment characterized mainly by elevated blood glucose levels. Diabetic foot ulcers (DFUs) significantly impact the patient’s quality of life and pose challenges for the healthcare system. Microalgae have emerged as effective agents for controlling inflammation owing to their elevated levels of active chemicals.

**Objectives:**

In the present study, we isolated and identified the *Haematococcus pluvialis* species of freshwater algae and investigated its effect on inhibiting the growth of diabetic wound bacteria.

**Methods:**

The ethanolic extract of this algae was investigated on antibiotic-resistant *Staphylococcus aureus* isolated from antibiotic-resistant wounds of diabetic patients. For this statistical study, a central composite design was used, considering factors such as gender, age, level of non-healing of the wound, and the effectiveness of the extract in inhibiting bacterial growth as the response.

**Results:**

The results showed that compared to other extracts, the ethanolic extract effectively inhibits bacterial growth up to 70% and 30%, respectively, of levofloxacin and ceftazidime antibiotics. The study of this extract with liquid chromatography-mass spectrometry led to the identification of the astaxanthin compound as the main component of the extract.

**Conclusions:**

This study has substantial prospects for future therapeutic uses and instills hope for enhanced therapies for DFUs.

## 1. Background

Over the last decades, we have faced a 30% increase in diabetes worldwide, which is expected to continue. Among the types of diabetes, type 2 diabetes is a disorder of the endocrine glands due to lifestyle factors and results in increased blood glucose levels due to insufficient or inappropriate insulin. Diabetes mellitus has increased from 1980 to 2014, and it is projected to increase by more than 50% by 2045 (1). The most important complications of type 2 diabetes include diabetic foot ulcers (DFUs), lower limb amputation, diabetic retinopathy and vision loss, atherosclerosis and thickening of the artery wall, and kidney and nerve damage. Although diabetes is costly, more than 70% of costs are related to diabetes-related disorders. More than 60% of DFUs lead to amputation every 30 seconds around the globe.

The etiology of chronic DFUs includes adhesion-associated medical devices, polymicrobial biofilms, impaired immune function, neuropathy, and inadequate blood supply, making the disease more complex (2). One of the most important consequences of the indiscriminate use of antibiotics is the formation of antibiotic-resistant bacteria with high growth rates and the transfer of genes for resistance (3). Diabetic ulcers are prone to infection by *Staphylococcus aureus* and *Pseudomonas* species, both of which can impede the healing of the affected tissue (4). These bacteria exhibit antibiotic resistance by forming biofilms and physical barriers, in addition to possessing certain genes (5).

Research aims to develop novel antibacterial agents with reduced adverse effects and enhanced efficacy, using herbal and natural substances for disease treatment, potentially reducing reliance on chemical pharmaceuticals or developing new lead molecules (6). Natural antibacterial agents, such as astaxanthin from marine ecosystems, have potential for medicinal use. The freshwater alga *Haematococcus pluvialis* is the richest natural source of this compound. Under stress conditions, including high light or nutrient starvation, the algae store high levels of astaxanthin, changing color from green to red. This natural protective mechanism renders *H. pluvialis* the top organism being raised for commercial production of astaxanthin. *Haematococcus pluvialis* contains antioxidants and flavonoids, potentially acting as an alternative to manufactured medicines. Research on this microalga could help treat DFUs, despite antibiotic-resistant microorganisms (7).

## 2. Objectives

The present study aims to isolate and identify the freshwater algae *H. pluvialis*, select the optimal solvent extraction method for active compound extraction, examine the inhibitory effects of these extracts on microorganisms linked to diabetic ulcers, and identify the bioactive chemicals using liquid chromatography-mass spectrometry.

## 3. Methods

### 3.1. Algae Separation and Identification

The study involved collecting aerated water samples from the Sefidroud River in Gujarat, Iran, where algal blooms were observed to isolate microalgae. A bold basal medium (BBM; Merck Co., Darmstadt, Germany) was autoclaved, and a 10 mL water sample was introduced into the medium. The flasks were subjected to continuous white fluorescent illumination for three weeks. Any growth was diluted with BBM, and subcultures were established by inoculating 50 mL onto Petri dishes with BBM solidified with 1.5% bacteriological agar (Agar: Merck Co., Darmstadt, Germany). The diluted samples were then introduced into a 96-well microtiter plate containing 200 mL of BBM. The cultures were incubated under continuous light for two weeks (8). Microscopic identification was carried out and later confirmed through molecular markers. The main criteria for algae identification were DNA, the NCBI GenBank database, and the phylogenetic tree.

### 3.2. DNA Extraction, PCR Amplification, Sequencing, and Phylogenetic Analysis

Algal cells were collected during the mid-to-late exponential growth phase (10 - 14 days) by centrifugation at 13,000 × g for 3 minutes at 4°C. Genomic DNA was extracted using a Plant Genomic DNA extraction kit (SolGent, Daejeon, S. Korea), following the manufacturer’s protocol (9). The DNA concentration was measured at 260 nm using a spectrophotometer (HACH, DR/4000v, USA). Amplification of the rDNA D1-D2 (LSU) coding region was performed using a T-Gradient thermocycler (Biometra GmbH, Gottingen, Germany). The ITS rDNA region was amplified using the universal primers ITS4 (5′-TCCTCCGCTTATTGATATGC-3’) (10). The specific gene accession number for *H. pluvialis* was MN933864. The PCR products were confirmed by agarose gel electrophoresis, purified using the Gel PCR Clean-Up System, and sequenced using a Dye Deoxy Terminator Cycle Sequencing Kit (Applied Biosystems, USA). An ABI Prism 377 DNA sequencer analyzed sequences from https://www.ncbi.nlm.nih.gov/nuccore/MN933864. The BLAST algorithm compared the ribosomal RNA gene sequences against the GenBank database. After manual inspection and alignment using Genedoc, sequences shorter than 200 nucleotides or longer than 1000 nucleotides, or those not belonging to green microalgae, were excluded. A phylogenetic tree was constructed using the neighbor-joining method with Kimura’s two-parameter model in the MEGA4 software package (11).

### 3.3. Extraction Process

The algal extract was prepared using ethanol, water, and ethyl acetate as solvents. The extraction was conducted at 25°C for 30 minutes with 900 watts of ultrasonic energy, with a 1:10 ratio W/V of algae to solvent. The amalgamation was subjected to an ultrasonic bath for 30 minutes. The extracts were filtered and dehydrated using a freeze-dryer. Concentration, patient gender, and health level were considered. The software suggested 200 treatment combinations, with selected values and levels shown in Table 1. All experiments were randomized to minimize uncontrolled variables. Bacterial content (OD590) was measured as the response in each experiment.

**Table 1.** Central Composite Design Factors and Factor Levels for Response Surface Statistical Design

Factor Types and Names; Codes	-α	-1	0	1	+α
**Quantitative factor**					
Age (y); A	35	40	55	65	75
Extract content (μg/mL); B	5	10	20	30	40
**Qualitative factor**					
Gender (a.u.); C					
Male	-	-	-	-	-
Female	-	-	-	-	-
Health level (a.u.); D					
Healthy (control)	-	-	-	-	-
Diabetes	-	-	-	-	-
Ulcer stage 1	-	-	-	-	-
Ulcer stage 2	-	-	-	-	-

### 3.4. Ulcer Sample Collection

The samples were provided by Physical Medicine and Rehabilitation Research Center in Tehran, Iran, and tested against *S. aureus* isolates from patients between 2022 and 2023. The study included participants aged 25 - 70, with 68% women and 32% men, and disease stage (4th stage of factor C). Exclusion criteria included cardiovascular diseases, gastrointestinal disorders, autoimmune issues, prolonged severe COVID-19 episodes, and mental health disorders.

### 3.5. Antibacterial Activity of Selected Ulcer Bacteria

Aerobic cultivation was carried out at 37°C and 450 rpm in Mueller-Hinton culture media. The anti-staphylococcus activity of algae was assessed using micro broth dilution methods according to NCCLS (12) protocols to determine each antibacterial agent’s minimum bactericidal concentration (MBC). The study involved preparing inoculant bacteria from freshly cultured *S. aureus* and diluting them in Mueller-Hinton broth (0.5 McFarland, the OD of 0.08 - 0.1 at 595 nm). The extract was prepared in concentrations ranging from 1 mg/mL to 1024 mg/mL in 96-well plastic microdilution plates. Minimum inhibitory concentrations were documented after a 22-hour incubation at 37°C. The antibacterial efficacy of the algal extract was assessed via the disc diffusion method. Overnight cultures of *S. aureus* were inoculated into normal saline and swabbed onto Mueller-Hinton agar media. Then, 100 μL of the algal extract was placed into a 6 mm well in the plate. The plates were incubated at 37°C for 24 hours, and inhibition zones were measured. All tests were performed in triplicate.

### 3.6. HPLC-MS Analysis of Algae Extract

The composition of the algae extract was analyzed using mass spectrometry and HPLC separation. The chromatographic process involved a binary pump and a C18 column with mobile phases A (methanol 85%; water 15%; 0.05% TFA) and B (acetonitrile 75%; dichloromethane 25%). The column was eluted with phase A, which increased to 75% in 5 minutes, followed by 0% in 30 minutes, and then reached 100% within 5 minutes. The flow rate was set to 200 nL/min. The Finigan LTQFT Ultra, equipped with a nano electro spray ionization (NESI) generator and an ion trap (IT) mass detector, was connected to the HPLC system for mass spectrometry analysis (Thermo Scientific Co., USA). The instrument’s control, data acquisition, and processing were handled by Xcalibur software. Under typical adverse ESI-MS conditions, a capillary voltage of 3.0 kV and a skimmer cone voltage of 30 V were applied to identify the metabolites (13).

## 4. Results

**Figure 1.**
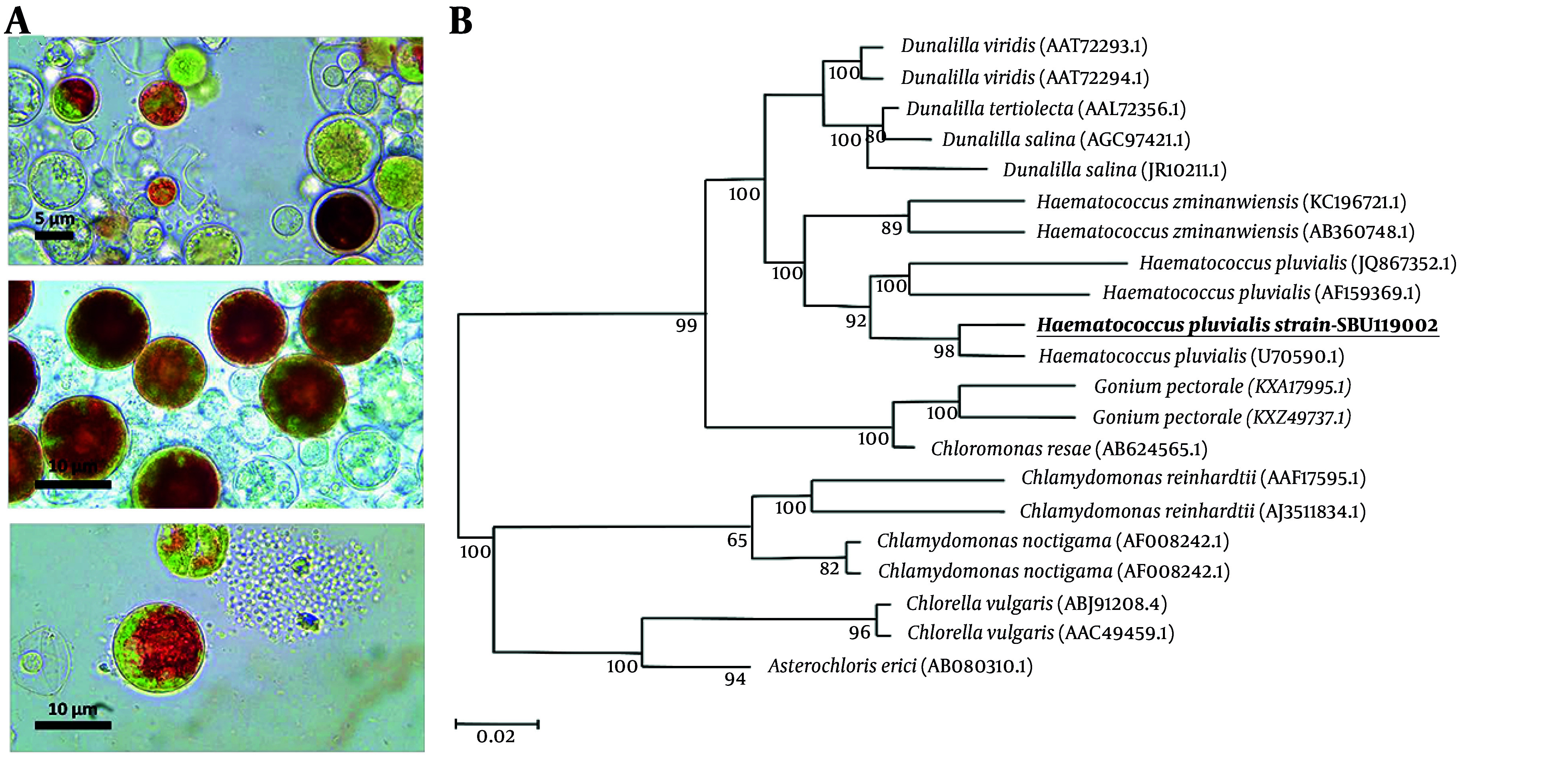
*Haematococcus pluvialis* A, light microscope images with 40 and 100 magnifications; B, phylogenetic tree generated by the neighbor-joining method based on partial 16S rRNA sequences showing the phylogenetic relationships between SBU isolates and closely related species of the genus. Bootstrap values (expressed as percentages of 1000 replications) are shown at significant branching points.

Four algal cultures were obtained from various sources, and a microalgae isolate was selected based on its cell morphology, dimensions, and ability to thrive. Microscopic examination confirmed the colonial structure and absence of contaminants (Figure 1). To verify the species, whole DNA and PCR-amplified rRNA (LSU) were extracted for molecular validation, addressing challenges in accurate microscopic identification due to the morphological diversity of algae. A study on eukaryotic algae used universal forward and reverse primers with PCR to amplify genomic DNA, resulting in a single amplified product for LSU rDNA (~ 850 bp in size). The LSU rRNA gene, which evolves more quickly than the SSU rRNA gene, can differentiate between closely related species through short diagnostic sequences. The study found that microalgae isolates shared 98% sequence similarity with *H. pluvialis*.

Algae extraction was performed using three solvents: Ethanol, water, and ethyl acetate. All three extracts were tested for their effectiveness against *S. aureus*, using antibiotics as a positive control and water as a negative control. The study used two methods: Minimum bactericidal concentration and the inhibition zone (Table 2) (14).

**Table 2.** Anti *Staphylococcu aureus* Minimum Biocidal Concentration (μg/ml) Determined for Different Algae Extracts Against Positive (Levofloxacin and Ceftazidime) and Negative (Water) Control

Extract and Control Conditions	*Staphylococcus aureus*			
	MBC (μg/mL)	STDEV	IZ	STDEV
***Haematococcus pluvialis***				
Ethanol EX.	16 ^a^	0.0311	2.87 ^a^	0.0109
Water EX.	512	0.0113	ND ^b^	0.0043
Ethyl acetate EX.	128	0.0230	1.55 ^a^	0.0090
**Positive control**				
Levofloxacin	4 ^a^	0.0122	3.08 ^a^	0.0111
Ceftazidime	8 ^a^	0.0176	2.98 ^a^	0.0312
**Negative control**				
Water	ND ^b^	ND ^b^	ND ^b^	ND ^b^

Abbreviations: MBC, minimum biocidal concentration; STDEV, standard deviations; IZ, inhibition zone.

^a^ growth inhibition effective concentration.

^b^ The MBC could not be determined due to detection in MBC higher concentrations.

Among the extracts, the algae ethanolic extract was the most active against bacteria and exhibited the highest antibacterial activity compared to the aqueous and ethyl acetate extracts. The aqueous extract showed minimal effect on either of the algal types (Table 2). 

The study investigated the efficacy of an ethanolic extract (Appendix 1 in Supplementary File) in inhibiting bacterial growth from patient wounds (Figure 3A, B, and C). The ethanolic extract of algae was tested for bacterial reduction under 595 nm light. The ANOVA findings (Table 3) showed that the most significant interaction was between patients’ age/health status and extract quantity/age. The model exhibited no systematic error, demonstrated adequate fit, and had R^2^ and adjusted R^2^ values close to or exceeding 85%.

**Table 3.** ANOVA for Response Surface 2FI Model Analysis of Variance Table ^a^

Sources	Sum of Squares	df	Mean Square	F-Value	P-Value (Prob > F)
**Model**	90.09	18	5.00	79.98	< 0.0001 ^b^
**A: Age**	0.35	1	0.35	5.58	0.0193 ^b^
**B: Extract content**	0.011	1	0.011	0.17	0.6776
**C: Gender**	0.13	1	0.13	2.12	0.1474
**D: Health level**	88.66	3	29.55	472.24	< 0.0001 ^b^
AB	0.039	1	0.039	0.62	0.0117 ^b^
AC	0.069	1	0.069	1.10	0.2947
AD	0.28	3	0.094	1.50	0.2161
BC	0.020	1	0.020	0.32	0.5746
BD	0.37	3	0.12	1.96	0.0011 ^b^
CD	0.16	3	0.053	0.85	0.4706
**Residual**	11.33	181	0.063	-	-
**Lack of fit**	3.63	53	0.068	1.14	0.2769
**Pure error**	7.70	128	0.060	-	-

^a^SD: 0.025; C.V.%: 16.18; R-squared: 88.83; Adj R-squared: 87.72.

^b^A P-value of ≤ 0.01 is considered statistically significant.

**Figure 2.**
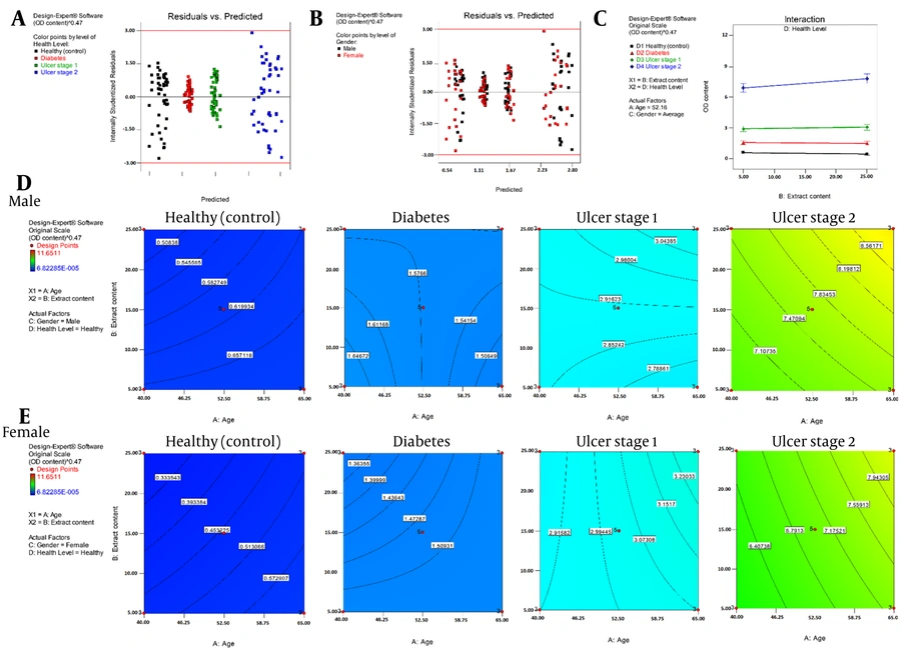
Statistical results of one-factor analysis against A, the illness and foot ulcer level; B, the gender of patients; and C, the *Haematococcus pluvialis* extract on the designed model. The surface response variations of the microbial content (OD) for the group of D, females and E, males at the specific levels of illnesses and foot ulcer

The study further investigated the efficacy of the ethanolic extract in inhibiting germ growth from patient wounds. The extract was tested for bacterial reduction under 595 nm light (Figure 2). The ANOVA findings showed that the most significant interaction was between the patient’s age/health status and extract quantity/age. The model exhibited no systematic error, demonstrated adequate fit, and had R^2^ and adjusted R^2^ values close to or exceeding 85%.

**Figure 3.**
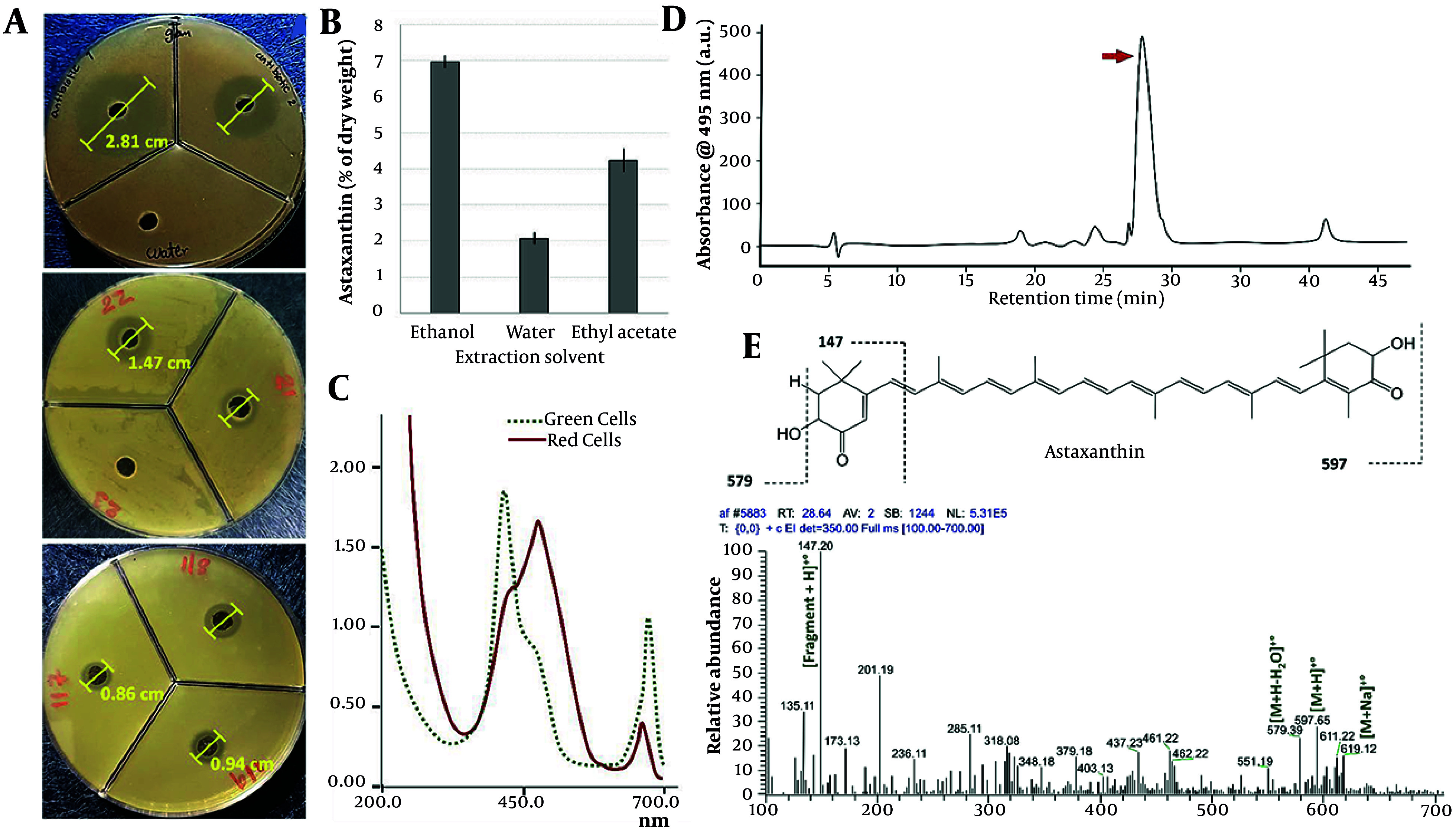
A, the inhibition zone results of ethanolic extracts of isolated *Haematococcus pluvialis* against ulcer-isolated *Staphylococcus aureus* (No. 21-22-23-117-118-119) compared to the antibiotics Ceftazidime (No. 1) and Levofloxacin (No. 2) as a positive control against water as a negative control. B, Astaxanthin content in dry extracta ;C, based on UV-Vis absorption spectrum; D, the HPLC-MS analysis of ethanolic extract; and E, the identified compound mass spectrum of Astaxanthin.

The ethanolic extract of algae was placed in a liquid chromatography-mass spectrometry apparatus (Figure 3D) for mass spectrometry testing. The analyzed material was of a separated type (Figure 3E). Compounds with a retention period of 28.64 minutes were separated via liquid chromatography, and their mass spectrometric spectra were acquired (Figure 3E). In the MS spectrum, the most important identified adduct ion peaks are [M+Na]^+•^, [M+H]^+•^, and [M+H-H_2_O]^+•^. Astaxanthin, containing the ions (Figure 3E) of [M+Na]^+•^: 619.12 m/z, [M+H]^+•^: 597.65 m/z, and [M+H-H_2_O]^+•^: 579.39 m/z, was identified as a separated peak at 28.64 minutes (Figure 3E) in the ethanolic extract (15, 16). These ions and fragments are suggested based on a study by the Metlin Online Library.

## 5. Discussion

Bacteria are the principal causative pathogens in diabetic foot infections, with global epidemiological data highlighting their pivotal role and resistance patterns (17). Their persistence is attributed to biofilm production and β-lactamase, which render antibiotic therapy challenging and underscore the need for novel treatment regimens (18). Natural products, including plant and algal extracts, have garnered interest due to their antioxidant and antimicrobial activity. Algal extracts and bioactive compounds such as astaxanthin, fucoidan, alginate, oleoresin, and phlorotannins have been considered significant natural resources in recent years to enhance the healing process and recuperation. Research has shown that algae, due to their complex saccharide, phenol, and bioactive content, have the potential to create a favorable wound environment for healing with antioxidant, anti-inflammatory, and antimicrobial action (19). These compounds exhibit properties that decrease the onset, suppress the growth of pathogenic microorganisms, trigger angiogenesis, and ultimately accelerate healing. Furthermore, algal compounds have been used as biocompatible dressings and hydrogels that create protective environments for storage (20). Most evidence comes from laboratory and animal trials, and clinical evidence is still limited, but the rising trend suggests they are likely to develop innovative products in wound care (21). For instance, *Bauhinia variegata* leaf extracts are highly antibacterial (6), while *H. pluvialis* oleoresin and other microalgal antioxidants have been discovered as promising sources of bioactivity (22). A genetic study on *H. pluvialis* revealed the primary pathways for astaxanthin biosynthesis (11). Apart from manufacturing, astaxanthin also has excellent antioxidant and antimicrobial activities, and algae extracts are promising against pathogenic bacteria (23). Combined, these findings position *H. pluvialis* as a multi-functional bioresource for therapeutics, nutraceuticals, and biotechnology, accompanied by analytical advancements in carotenoid quantification and species verification (15).

The research involved the isolation and identification of *H. pluvialis* freshwater algae species, and its extracts were tested for antibacterial activity against bacteria from DFUs. The ethanolic extract showed antibacterial efficacy analyzed through LC-MS. The main identified compound is astaxanthin, which may be responsible for its antibacterial properties (15). The results indicate that three tests employing Std codes 21, 22, 117, 118, and 119 had maximum inhibition of bacterial growth and minimum optical density (OD) at 48 hours for *H. pluvialis*. The meticulous statistical elaboration of the data obtained (Figure 2A) validated a significant effect of the laboratory results on the percentage of inhibition of bacterial growth. Therefore, the bacteria isolated from the ulcers in non-diabetic patients exhibit the maximum inhibition rate. According to the study, bacterial inhibition in DFUs is minimal among those patients who are not responsive to standard therapeutic approaches. The model validity is low during the ulcer phase (Figure 2). 

### 5.1. Conclusions

Microalgae extract can help digest and rectify ulcers, colitis, Crohn’s disease, and diverticulosis by supporting beneficial bacteria in the digestive tract (7). Microalgae ethanolic extract exhibits potent antibacterial activity by inhibiting bacterial growth in diabetic wounds. Natural extracts, like algal extract compounds, exert multiple beneficial effects on diabetes. Enhanced insulin sensitivity is accompanied by less oxidative stress in pancreatic β-cells. Regulation of this process is crucial in the regulation of blood glucose levels. Therefore, algal natural compounds can be used as a useful supplement for type 2 diabetics. In this context, it was noted that the ethanolic extract of *H. pluvialis* has suitable antibacterial activity by inhibiting the growth of microorganisms associated with diabetic ulcers and contains astaxanthin. This investigation examines the potential antibacterial activity of natural compounds but does not claim to provide an exhaustive or completely accurate evaluation, especially against resistant bacteria. It was not performed on wounds and is not sound enough for immediate application in humans. More precise human studies are recommended before any practical use. Within these limits, the statistical results are sufficient.

ijpr-24-1-161297.pdf

## Data Availability

The processed data used and analyzed during the present study are available from the corresponding author upon reasonable request.
